# Spatial and temporal evolution of Lassa virus in the natural host population in Upper Guinea

**DOI:** 10.1038/srep21977

**Published:** 2016-02-25

**Authors:** Elisabeth Fichet-Calvet, Stephan Ölschläger, Thomas Strecker, Lamine Koivogui, Beate Becker-Ziaja, Amara Bongo Camara, Barré Soropogui, N’Faly Magassouba, Stephan Günther

**Affiliations:** 1 Bernhard Nocht Institute for Tropical Medicine, Hamburg, Germany; 2 Institute of Virology, Philipps University Marburg, Marburg, Germany; 3 Projet des Fièvres Hémorragiques en Guinée, Hopital Donka, Conakry, Guinea

## Abstract

This study aimed at reconstructing the spatial and temporal evolution of Lassa virus (LASV) in the natural host population. To this end, we generated 132 partial nucleoprotein sequences of LASV from *M. natalensis* trapped in 12 villages around Faranah, Upper Guinea, over a period of 12 years. This study reveals two main features of LASV evolution in *M. natalensis*. First, the virus evolves in the reservoir with a molecular clock rate of 9 (7–11) × 10^–4^ position^–1^ year^–1^ implying that contemporary LASV lineages circulate in the Faranah area since less than 100 years. Second, viruses circulating in a specific village are diverse and polyphyletic. We observed, however, there are monophyletic clusters at village and sub-village level at specific points in time. In conclusion, our data indicate that the temporal and spatial pattern of LASV evolution in the natural reservoir is characterized by a combination of stationary circulation within a village and virus movement between villages. The latter feature is relevant for rodent control strategies, as it implies that recurrence of the virus from neighbouring villages may occur in villages where the virus has previously been eradicated.

Lassa virus (LASV) was discovered in 1969 in Nigeria^1^. The virus is estimated to cause about 200,000 infections with up to 5,000 deaths each year in West Africa^2^. The disease caused by LASV, Lassa fever, is endemic in two distinct endemic areas, one in Nigeria and the second in Sierra Leone-Guinea-Liberia-Mali^3^. It is transmitted to humans through the multimammate mouse, *Mastomys natalensis*, which is living commensally in rural areas of West Africa^4^,^5^.

LASV is a member of the family *Arenaviridae* containing two genome segments of single-stranded RNA. The small RNA segment (S-RNA) encodes the nucleoprotein (NP) and the glycoprotein precursor (GPC). The large RNA segment (L-RNA) encodes the RNA-dependent RNA polymerase (L) and the matrix protein Z^6^. Phylogenetic analyses showed that LASV currently consists of five lineages, with three lineages found in Nigeria, one in Sierra Leone, Guinea and Liberia, and one in Mali and Côte d’Ivoire^7^,^8^,^9^. Geographical pattern of the lineages indicates that the virus has spread from east to west^7^. Timing of the phylogenetic divergence events in LASV evolution depends on estimation of the molecular clock rate of the virus. So far, studies estimating the substitution rate mainly relied on LASV sequences obtained from human infections scattered over a large geographic area. In this study, we aimed at reconstructing the spatial and temporal evolution of LASV in the natural host population. To this end, we generated 132 partial LASV sequences from *M. natalensis* trapped in 12 villages of the Faranah prefecture in Upper Guinea over a period of 12 years (Fig. 1). As the longitudinal survey is conducted in a specific geographical area, the sequences obtained are likely to represent a sample from the transmission chains among the local rodent population and are thus suitable to estimate both the short-term evolutionary rate of LASV in the natural reservoir as well as the timing of virus spread of LASV at regional level.

## Results

To search for a temporal signal in our dataset of 132 LASV NP sequences, a phylogenetic tree was inferred using a maximum likelihood approach and subjected to root-to-tip regression analysis. This test revealed a temporal structure in the data with statistically significant correlation between date of sampling and root-to-tip divergence (R^2^ = 0.22, p < 0.005, n = 132). The slope of the regression line suggested that the ancestors of the contemporary LASV strains emerged around 1925 in the area (x-intercept = 1924.9, Fig. 2).

For time-scaled phylogenetic reconstruction, we first aimed to determine the models that fit best to our data. The program FindModel identified the general time-reversible model of sequence evolution with a gamma distribution of among-site nucleotide substitution rate variation (GTR + gamma) as best-fit substitution model. In addition, we compared 8 different models using a Bayesian approach (Table S1). According to Akaike’s information criterion through Markov chain Monte Carlo (AICM), constant population size fitted best with both strict and relaxed clock models. The coefficient of variation for the relaxed clock models was small with a median of 0.13, indicating that the branches show only minor differences in the molecular clock rate (Figs. S1 and S2). This suggests that the data are clock-like and a strict clock model is appropriate for phylogenetic reconstruction^10^. Eventually, we estimated substitution rate and dates of divergence events under the assumption of GTR + gamma and constant population size with strict and relaxed clock and with and without codon partitioning (Table 1). The model settings hardly influenced the estimates indicating a robust reconstruction. All models estimated a substitution rate of 9 × 10^–4^ position^–1^ year^–1^ (Highest Posterior Density interval containing 95% of the posterior probability distribution [95% HPD]: 7–11 × 10^–4^ position^–1^ year^–1^) and an age of the most recent common ancestor (MRCA) of 83–86 years. The latter corresponds well with the age determined by root-to-tip regression.

The phylogenetic tree reconstructed with GTR + gamma, constant population size, strict clock, and codon partitioning features three well supported main clades (posterior probability >0.85) called “Faranah I”, “Faranah II “ and “Faranah III” (Fig. 3). Faranah I includes only two sequences (nos 20 and 24) obtained in Yarawalia, November 2013. They branch directly at the root. Faranah II includes ten sequences obtained from *M. natalensis* trapped in Bantou (nos. 375, 377, and 390), Dalafilani (no. 04), Damania (nos. 05, 90 and 95) and Sokourala (nos. 61, 75 and 76). Sequences obtained in Bantou in September 2003 have never been sampled again despite five extensive trapping sessions in the same village between 2003 and 2011. Sequence analysis of repeated viral RNA extraction from both spleen and liver confirmed the unique sequences. According to the molecular clock analysis, the Faranah II clade emerged 55–56 years before 2014 (Table 1). Clade Faranah III comprises the remaining sequences from all localities studied. It includes the sequences from Bantou, Brissa, Dalafilani, Damania, Gbetaya, Safrani, Sokourala, Sonkonia and Yarawalia in a well-supported sub-clade, and those from Tanganya, Gbetaya, Khoria and Yarawalia in a poorly supported sub-clade. The MRCA of all Faranah III sub-clades seems to be 45–46 years old (Table 1). While there is sub-clustering of strains at the village level, none of the sequences from a specific village form a monophyletic group in the phylogeny — they are all polyphyletic — indicating multiple movements of viruses between villages. We have visualized the virus spread in the study area by phylogeographic reconstruction (Fig. S3). A similar pattern is observed at the level of trapping lines within each village. Although sequences obtained from animals trapped at the same line often cluster together, none of the trapping lines forms a monophyletic cluster. For example, Bantou line #14 is monophyletic for the 2003 trapping session, but becomes paraphyletic if the trapping of 2004 is included in the analysis (then one 2004 sequence from line #10 clusters with the 2003 line #14 sequences). Similarly, the Bantou line #10 (a maize field near the village) is monophyletic for sequences sampled in 2003 (no. 375, 377, and 390), but becomes polyphyletic with the sequences trapped at this line in 2004. Thus, at the sub-village level there is evidence of monophyly at single points in time, but over time the spatial segregation is obliterated.

## Discussion

This study reveals two main features of LASV evolution in *M. natalensis*. First, the virus evolves in the reservoir with a molecular clock rate between 7–11 × 10^–4^ position^–1^ year^–1^, which is similar to previous estimations based on other arenavirus sequences or LASV sequences obtained from humans^11^,^12^. Second, viruses circulating in a specific locality are diverse and polyphyletic with respect to viruses from neighbouring villages. However, there are monophyletic clusters formed by viruses from a village at specific points in time, indicating that the temporal and spatial pattern of LASV evolution in the natural reservoir is characterized by a combination of stationary circulation within a village and virus movement between villages. The latter feature is relevant for rodent control strategies, as it implies that in villages where the virus has been eradicated by rodent control measures, a repopulation from neighbouring villages is possible. Thus, for rodent control to be effective, such programs should be conducted at a larger geographic scale and sustained over a prolonged period of time. This conclusion is supported by a recent study of LASV diversity in rodents in Sierra Leone showing that one sequence from the village Saama, north to Kenema, clusters with Barlie, a village 60 km apart^13^.

The observed spatial and temporal pattern in LASV evolution might be explained by the territorial behaviour of *M. natalensis*. Recently, Borremans *et al.*^14^ have shown that *M. natalensis* is a non-territorial species, with individuals extensively sharing their home range of approximately 600 m^2^ with congeners. Home ranges overlap regardless of age and sex of individuals as well as season. This behaviour in particular, combined with a high horizontal transmission rate within the rodent population^15^, explains the lack of spatial clustering of virus sequences at the sub-village level (trapping lines). It may also contribute to the spread of virus between villages, though the distance between villages exceeds the home range of the animals. Therefore, long-range movement of virus between villages and thus also the propagation of the human disease may be facilitated by human behaviour. An obvious factor could be transport of infected *M. natalensis* with goods and foodstuff. However, the virus may also spread through the transport of contaminated food accessed by *M. natalensis* at the new location. Finally, it may not be excluded that virus spreads through the movement of humans who recovered from Lassa fever and initiate a reverse zoonosis via excretion of still infectious body fluids.

Our analysis suggests that the MRCA of the contemporary LASV strains has been introduced in the Faranah area less than 100 years ago. However, this estimate has to be interpreted with caution. We have sampled not all villages of the area potentially causing a sampling bias. Older lineages such as Faranah I , which was found only in one village (Yarawalia) and once in time, may have escaped detection. On the other hand, lack of more recent sequences for well-supported lineages may point to an important scenario in LASV evolution: extinction. For example, despite intense follow-up trapping, we have never sampled again sequences belonging to one Bantou lineage formed by sequences nos. 375, 377, and 390 from 2003 in Faranah II. If phylogenetically “older” lineages got extinct and therefore do not appear in the sequence sample, the age of the MRCA of the Faranah clade does not correspond to actual time of virus circulation in the area. Evolution of LASV in circles of generation, spatial movement, and extinction of lineages would imply its presence in West Africa for longer time than the analysis of contemporary sequences suggests. Long-term longitudinal studies are warranted to gain further insights into these aspects.

## Methods

**Figure 1:**
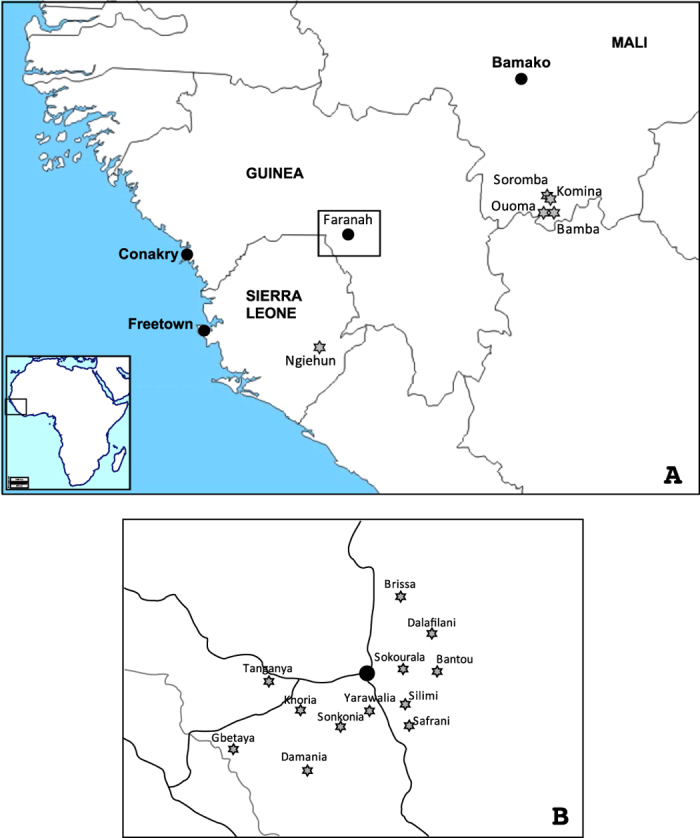
A-Map of Guinea, Sierra Leone and Mali showing the origin of LASV strains obtained in the natural host, *Mastomys natalensis*. The maps of Africa and West Africa were downloaded from http://d-maps.com/carte.php?num_car=728&lang=fr and http://d-maps.com/carte.php?num_car=752&lang=fr, and then modified in using the software EazyDraw v 5.3.0 (http://eazydraw.com). B- Detailed location of the 12 villages around Faranah: Bantou (10°3.586′N; 10°35.169′W), Brissa (10°13.010′N; 10°41.326′W), Dalafilani (10°08.590′N; 10°36.303′W), Damania (09°48.410′N; 10°51.796′W), Gbetaya (09°50.461′N; 11°2.409′W), Khoria (09°56.536′N; 10°53.582′W), Safrani (10°03.469′N; 10°44.302′W), Silimi (09°58.609′N; 10°49.173′W), Sokourala (10°03.407′N; 10°39.950′W), Sonkonia (09°54.763′N; 10°47.888′W), Tanganya (10°0.024′N; 10°58.367′W), Yarawalia (09°57.267′N; 10°43.913′W). The map has been drawn in EazyDraw v 5.3.0 (http://eazydraw.com), based on the geographical coordinates generated in an Excel chart.

**Figure 2:**
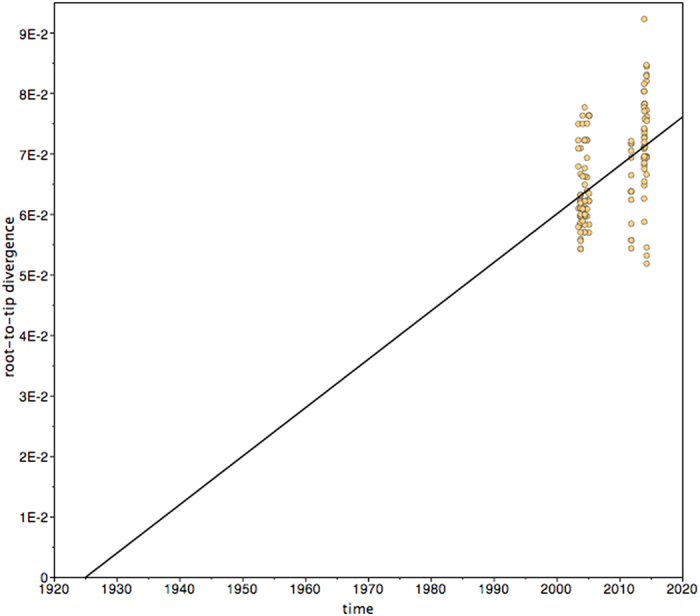
Root-to-tip distance versus collection date of the 132 LASV NP sequences. A phylogenetic tree was inferred by a maximum likelihood method without molecular clock and the genetic distance from the root to the tip of the branches was correlated with the corresponding trapping dates. The regression analysis revealed a positive correlation between genetic distance and trapping date with R^2^ = 0.22 (p < 0.005). The intercept of the regression line with the x-axis reflects the date at the root of the tree, i.e. the time of the MRCA.

**Figure 3:**
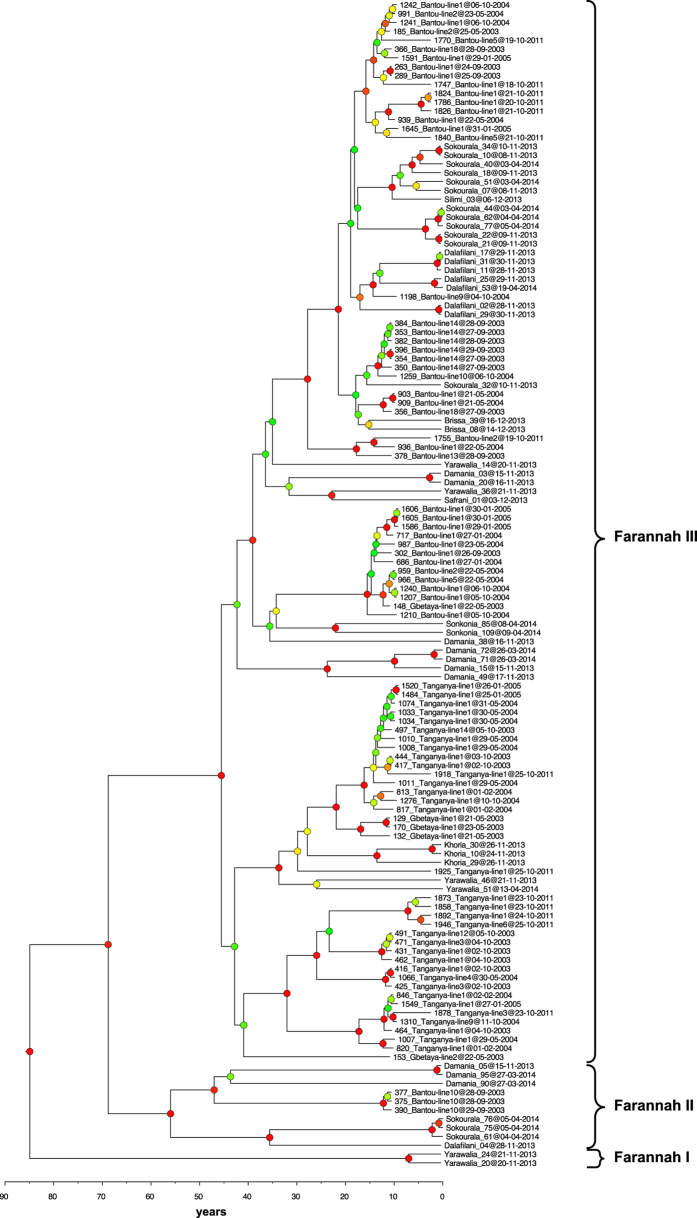
Phylogenetic analysis of LASV NP sequences from 132 *Mastomys natalensis* trapped during 12 years. The day of capture, the village, and the trapping line is indicated for each sequence. The tree, based on 754-nucleotide fragment of the NP gene, was inferred by using the Bayesian Markov Chain Monte Carlo method, with GTR + gamma model, strict clock, constant population size and partitioning into codon positions 1,2,3. Posterior probabilities are coded as follows: <0.50, green dots; 0.50 to <0.80, yellow dots; 0.80 to <0.95, orange dots; >0.95, red dots.

The study was conducted from May 2003 to April 2014. From May 2003 to January 2005, two villages —Bantou and Tanganya (Fig. 1) — were visited 3 times per year to perform a longitudinal survey in the rodent population. To increase the temporal and spatial scale of the study, complementary trapping expeditions were performed in October 2011, and in 2013-2014 in Brissa, Dalafilani, Damania, Khoria, Safrani, Silimi, Sokourala, Sonkonia and Yarawalia, (Fig. 1). Gbetaya was investigated once in May 2003. Traps (Sherman Traps, Inc.) were set in different habitats: houses, cultivations, savannah, forest during 3 consecutive nights. Inside the houses, 100–120 traps were set along a transect (=lines) crossing the village with 2 traps per room. Outside the houses, 100–220 traps were set along 5–11 transects of 100 m in length with 1 trap each 5 m^16^. The trapping effort for all missions represented a total of 19,535 trap-nights. Approval for the investigation was obtained from the National Ethics Committee of Guinea (permit n° 2003/PFHG/05/GUI and 12/CNERS/12). The methods were carried out in accordance with the approved guidelines.

Rodent specimens were first screened for the presence of arenaviruses following the protocol described in Vieth *et al.*^17^. From LASV positive samples, a fragment of 800 nucleotides within the NP coding region was amplified using a nested RT-PCR protocol with the following primers: outer primers LVS 1607-fwd (GGTGTTGATGTTCTAAASACC) and LVS 2535-rev (GCCTGCATGTTGGATGGTGGC); nested primers LVS 1629-fwd (TGTCTCTGGGCAGCACTGCTC) and LVS 2429-rev (TGTTTGTCTCAGACACTCCYGGTG). Remaining gaps were closed after designing strain-specific primers. The amplified NP region corresponds to the one used by Bowen *et al.*^7^ for phylogenetic analysis of LASV. PCR fragments were sequenced on both strands. The sequences were assembled and aligned by using Mac Vector software (2011 Mac Vector, Inc). In total, we generated 132 partial NP sequences (754 nucleotides) of LASV from *M. natalensis* from Gbetaya (n = 5 sequences), Bantou (n = 45), Tanganya (n = 34), Brissa (n = 2), Dalafilani (n = 8), Damania (n = 10), Khoria (n = 3), Safrani (n = 1), Silimi (n = 1), Sokourala (n = 15), Sonkonia (n = 2), and Yarawalia (n = 6). Each sequence was time-calibrated with the day of capture (Table S2). Sequences were submitted to GenBank under accession numbers KP339050-KP339133 and KT833175- KT833222.

The sequences were aligned and subjected to time-scaled phylogenetic analysis. The program FindModel (http://www.hiv.lanl.gov/content/sequence/findmodel/findmodel.html) identified the general time-reversible model of sequence evolution with a gamma distribution of among-site nucleotide substitution rate variation (GTR + gamma) as the substitution model that best describes the data set. Phylogeny was inferred by the Bayesian Markov Chain Monte Carlo (MCMC) method implemented in BEAST software^18^. The analysis was performed using various assumptions and model settings to evaluate the robustness of the estimations inferred from the models. The following settings were used: GTR + gamma, strict and relaxed clock (uncorrelated lognormal); non-partitioning and partitioning into codon positions “1,2,3”; and population growth models: constant size, exponential growth, logistic growth and Bayesian skyline. MCMC chains were run for 100 million states, and sampled every 10,000 or 100,000 states to obtain an effective sample size above 200 for all the parameters (Table 1). Models were compared using a posterior simulation-based analogue of Akaike’s information criterion through MCMC (AICM)^19^. The comparison was conducted in Tracer v1.6 using log files containing 1001 states with 10% burn-in (Table S1). The substitution rate was estimated from the data without prior.

A phylogeographic model with discrete diffusion was reconstructed using BEAST with the following settings: GTR + gamma, constant population size, strict clock, and villages as traits. A root-to-tip regression analysis was performed with Path-O-Gen software to investigate the temporal signal in the data (http://tree.bio.ed.ac.uk/software/pathogen/). Phylogeny without a molecular clock was inferred by the maximum likelihood method implemented in PHYML^20^, and then analysed in Path-O-Gen with the dated tips.

## Additional Information

**How to cite this article**: Fichet-Calvet, E. *et al.* Spatial and temporal evolution of Lassa virus in the natural host population in Upper Guinea. *Sci. Rep.* **6**, 21977; doi: 10.1038/srep21977 (2016).

## Supplementary Material

Supplementary Information

## Figures and Tables

**Table 1:** Substitution rate of LASV in *M. natalensis* and age of main clades.

Model	Molecular clock/codon partition	AICM value	Substitution rate, median (95% HPD) [mutation x position^–1^ year^–1^]	Root age, median (95% HPD) [years]	Faranah II cluster age, median (95% HPD) [years]	Faranah III cluster age, median (95% HPD) [years]
#1	Strict/no partition	11836	8.93 (6.70–11.35) × 10^–4^	83.5 (60.1–113.2)	55.3 (41.4–73.3)	44.7 (34.3–57.4)
#2	Strict/codons 1, 2, 3	11523	8.91 (6.78–11.25) × 10^–4^	84.9 (60.7–114.0)	56.0 (41.1–73.8)	45.5 (35.3–58.0)
#3	Relaxed lognormal/no partition	11911	8.89 (6.67–11.43) × 10^–4^	84.7 (57.4–119.0)	56.1 (39.3–76.2)	45.7 (34.5–59.4)
#4	Relaxed lognormal/codons 1, 2, 3	11535	8.89 (6.42–11.31) × 10^–4^	86.2 (59.4–126.3)	56.4 (39.2–77.3)	46.0 (34.0–60.0)

Analysis was performed using GTR + gamma and constant population size.

Abbreviations: 95% HPD, Highest Posterior Density interval contains 95% of the posterior probability distribution; AICM, model evaluation using Akaike’s information criterion through Markov chain Monte Carlo. Lower values indicate a better fit to the data.

## References

[b1] Frame J. D., Baldwin J. M. J., Gocke D. J. & Troup J. M. Lassa fever, a new virus disease of man from West Africa. Am. J. Trop. Med. Hyg. 19, 670–676 (1970). 4246571 10.4269/ajtmh.1970.19.670

[b2] McCormick J. B. & Fisher-Hoch S. P. Lassa fever. Curr. Top. Microbiol. Immunol. 262, 75–109 (2002). 11987809 10.1007/978-3-642-56029-3_4

[b3] Fichet-Calvet E. & Rogers D. J. Risk maps of Lassa fever in West Africa. PLoS Neglected Tropical Diseases 3, e388, doi: 10.1371/journal.pntd.0000388 (2009). 19255625 PMC2644764

[b4] Lecompte E. *et al.* *Mastomys natalensis* and Lassa fever, West Africa. Emerg. Infect. Dis. 12, 1971–1974 (2006). 17326956 10.3201/eid1212.060812 PMC3291371

[b5] Monath T. P., Newhouse V. F., Kemp G. E., Setzer H. W. & Cacciapuoti A. Lassa virus isolation from *Mastomys natalensis* rodents during an epidemic in Sierra Leone. Science 185, 263–265 (1974). 4833828 10.1126/science.185.4147.263

[b6] Gunther S. & Lenz O. Lassa virus. Crit Rev Clin Lab Sci 41, 339–390 (2004). 15487592 10.1080/10408360490497456

[b7] Bowen M. D. *et al.* Genetic diversity among Lassa virus strains. J. Virol. 74, 6992–7004 (2000). 10888638 10.1128/jvi.74.15.6992-7004.2000 PMC112216

[b8] Manning J. T., Forrester N. & Paessler S. Lassa virus isolates from Mali and the Ivory Coast represent an emerging fifth lineage. Front. Microbiol. 6, 1037, doi: 10.3389/fmicb.2015.01037 (2015). 26483768 PMC4589679

[b9] Kouadio L. *et al.* Lassa Virus in Multimammate Rats, Cote d’Ivoire, 2013. Emerg. Infect. Dis. 21, 1481–1483, doi: 10.3201/eid2108.150312 (2015). 26196447 PMC4517737

[b10] Drummond A. J. & Bouckaert R. R. Bayesian Evolutionary Analysis with BEAST. (Cambridge University Press, 2015).

[b11] Coulibaly-N’Golo D. *et al.* Novel arenavirus sequences in *Hylomyscus sp*. and *Mus* (*Nannomys*) *setulosus* from Cote d’Ivoire: implications for evolution of arenaviruses in Africa. PLoS ONE 6, e20893, doi: 10.1371/journal.pone.0020893 (2011). 21695269 PMC3111462

[b12] Ehichioya D. U. *et al.* Current molecular epidemiology of Lassa virus in Nigeria. J. Clin. Microbiol. 49, 1157–1161, doi: 10.1128/JCM.01891-10 (2011). 21191050 PMC3067713

[b13] Leski T. A. *et al.* Sequence variability and geographic distribution of Lassa virus, Sierra Leone. Emerg. Infect. Dis. 21, 609–618, doi: 10.3201/eid2104.141469 (2015). 25811712 PMC4378485

[b14] Borremans B. *et al.* Happily together forever: temporal variation in spatial patterns and complete lack of territoriality in a promiscuous rodent. Population Ecology 56, 109–118 (2014).

[b15] Fichet-Calvet E., Becker-Ziaja B., Koivogui L. & Gunther S. Lassa serology in natural populations of rodents and horizontal transmission. Vector Borne Zoonotic Dis. 14, 665–674, doi: 10.1089/vbz.2013.1484 (2014). 25229705 PMC4170823

[b16] Fichet-Calvet E. *et al.* Fluctuation of abundance and Lassa virus prevalence in Mastomys natalensis in Guinea, West Africa. Vector Borne Zoonotic Dis. 7, 119–128, doi: 10.1089/vbz.2006.0520 (2007). 17627428

[b17] Vieth S. *et al.* RT-PCR assay for detection of Lassa virus and related Old World arenaviruses targeting the L gene. T. Roy. Soc. Trop. Med. H. 101, 1253–1264 (2007). 10.1016/j.trstmh.2005.03.018 17905372

[b18] Drummond A. J. & Rambaut A. BEAST: Bayesian evolutionary analysis by sampling trees. BMC Evolutionary Biology 7, 214 (2007). 17996036 10.1186/1471-2148-7-214 PMC2247476

[b19] Baele G. *et al.* Improving the accuracy of demographic and molecular clock model comparison while accommodating phylogenetic uncertainty. Mol Biol Evol 29, 2157–2167, doi: 10.1093/molbev/mss084 (2012). 22403239 PMC3424409

[b20] Guindon S. *et al.* New algorithms and methods to estimate maximum-likelihood phylogenies: assessing the performance of PhyML 3.0. Syst Biol 59, 307–321, doi: 10.1093/sysbio/syq010 (2010). 20525638

