# Supplementary material for: Spatial and temporal evolution of Lassa virus in the natural host population in Upper Guinea
Source: Sci Rep. 2016 Feb 25;6:21977. doi: 10.1038/srep21977 (PMC4766397; doi:10.1038/srep21977)
Supplement: Supplementary Information [file srep21977-s1.pdf]

Supplementary information

Title: Spatial and temporal evolution of Lassa virus in the natural host population in Upper Guinea

Authors: Elisabeth Fichet-Calvet[1*], Stephan Ölschläger[1], Thomas Strecker[2], Lamine Koivogui[3], Beate Becker-Ziaja[1], Amara Bongo Camara[3], Barré Soropogui[3], N'Faly Magassouba[3] and Stephan Günther[1]

Table S1: Comparison of different models using Akaike's information criterion through Markov chain Monte Carlo (AICM). Lower values of marginal likelihood indicate a better fit to the data. The best-fit model is indicated in bold.

	Settings		AICM value	
Model	Clock	Population	+/– standard error	
**#1**	**Strict**	**Constant**	**11836**	**+/– 0.54**
#5	Strict	Exponential	11866	+/– 0.48
#6	Strict	Logistic	11861	+/– 0.81
#7	Strict	Skyline	11865	+/– 0.45
#3	Relaxed	Constant	11911	+/– 0.65
#8	Relaxed	Exponential	11913	+/– 0.86
#9	Relaxed	Logistic	11923	+/– 1.10
#10	Relaxed	Skyline	11933	+/– 1.11

Analysis was performed using GTR+gamma without codon partitioning.

Table S2: List of the 132 LASV positive *M. natalensis* included in the study with trapping date, trapping site (village, habitat, and line), sex, and GenBank accession number of the LASV sequence (ID). For animals trapped outside of houses, the distance from the trapping site to the nearest house (dist) is provided.

N°viro	Village	date	sex	habitat	line	dist	ID
129	Gbetaya	21-May-2003	f	houses	line 1		KP339050
132	Gbetaya	21-May-2003	f	houses	line 1		KP339051
148	Gbetaya	22-May-2003	m	houses	line 1		KP339052
153	Gbetaya	22-May-2003	m	houses	line 1		KP339053
170	Gbetaya	23-May-2003	f	cultivation and bananas	line 2	150 m	KP339054
185	Bantou	25-May-2003	f	cultivation and orchard	line 2	700 m	KP339055
263	Bantou	24-Sep-2003	m	houses	line 1		KP339056
289	Bantou	25-Sep-2003	m	houses	line 1		KP339057
302	Bantou	26-Sep-2003	m	houses	line 1		KP339058
350	Bantou	27-Sep-2003	f	maize	line 14	60 m	KP339059
353	Bantou	27-Sep-2003	f	maize	line 14	60 m	KP339060
354	Bantou	27-Sep-2003	m	maize	line 14	60 m	KP339061
356	Bantou	27-Sep-2003	m	maize	line 18	100 m	KP339062
366	Bantou	28-Sep-2003	m	maize	line 18	100 m	KP339063
375	Bantou	28-Sep-2003	f	maize	line 10	50 m	KP339064
377	Bantou	28-Sep-2003	f	maize	line 10	50 m	KP339065
378	Bantou	28-Sep-2003	m	maize	line 13	50 m	KP339066
382	Bantou	28-Sep-2003	f	maize	line 14	60 m	KP339067
384	Bantou	28-Sep-2003	f	maize	line 14	60 m	KP339068
390	Bantou	29-Sep-2003	f	maize	line 10	50 m	KP339069
396	Bantou	29-Sep-2003	m	maize	line 14	60 m	KP339070
416	Tanganya	2-Oct-2003	f	houses	line 1		KP339071
417	Tanganya	2-Oct-2003	f	houses	line 1		KP339072
425	Tanganya	2-Oct-2003	f	maize and ochra	line 3	30 m	KP339073
431	Tanganya	2-Oct-2003	m	houses	line 1		KP339074
444	Tanganya	3-Oct-2003	m	houses	line 1		KP339075
462	Tanganya	4-Oct-2003	m	houses	line 1		KP339076
464	Tanganya	4-Oct-2003	f	houses	line 1		KP339077
471	Tanganya	4-Oct-2003	m	maize and ochra	line 3	30 m	KP339078
491	Tanganya	5-Oct-2003	m	wooded fallowland	line 12	80 m	KP339079
497	Tanganya	5-Oct-2003	f	grassland and pasture	line 14	50 m	KP339080
686	Bantou	27-Jan-2004	f	houses	line 1		KP339081
717	Bantou	27-Jan-2004	m	houses	line 1		KP339082
813	Tanganya	1-Feb-2004	f	houses	line 1		KP339083
817	Tanganya	1-Feb-2004	m	houses	line 1		KP339084
820	Tanganya	1-Feb-2004	m	houses	line 1		KP339085

846	Tanganya	2-Feb-2004	f	houses	line 1		KP339086
903	Bantou	21-May-2004	m	houses	line 1		KP339087
909	Bantou	21-May-2004	f	houses	line 1		KP339088
936	Bantou	22-May-2004	f	houses	line 1		KP339089
939	Bantou	22-May-2004	m	houses	line 1		KP339090
959	Bantou	22-May-2004	f	cultivation/fallowland	line 2	50 m	KP339091
966	Bantou	22-May-2004	m	cultivation/fallowland	line 5	30 m	KP339092
987	Bantou	23-May-2004	f	houses	line 1		KP339093
991	Bantou	23-May-2004	m	cultivation/fallowland	line 2	50 m	KP339094
1007	Tanganya	29-May-2004	f	houses	line 1		KP339095
1008	Tanganya	29-May-2004	f	houses	line 1		KP339096
1010	Tanganya	29-May-2004	f	houses	line 1		KP339097
1011	Tanganya	29-May-2004	m	houses	line 1		KP339098
1033	Tanganya	30-May-2004	m	houses	line 1		KP339099
1034	Tanganya	30-May-2004	m	houses	line 1		KP339100
1066	Tanganya	30-May-2004	m	cultivation and orchard	line 4	40 m	KP339101
1074	Tanganya	31-May-2004	m	houses	line 1		KP339102
1198	Bantou	4-Oct-2004	m	cultivation/fallowland	line 9	50 m	KP339103
1207	Bantou	5-Oct-2004	m	houses	line 1		KP339104
1210	Bantou	5-Oct-2004	m	houses	line 1		KP339105
1240	Bantou	6-Oct-2004	m	houses	line 1		KP339106
1241	Bantou	6-Oct-2004	f	houses	line 1		KP339107
1242	Bantou	6-Oct-2004	m	houses	line 1		KP339108
1259	Bantou	6-Oct-2004	m	maize	line 10	50 m	KP339109
1276	Tanganya	10-Oct-2004	f	houses	line 1		KP339110
1310	Tanganya	11-Oct-2004	m	maize	line 9	30 m	KP339111
1484	Tanganya	25-Jan-2005	f	houses	line 1		KP339112
1520	Tanganya	26-Jan-2005	m	houses	line 1		KP339113
1549	Tanganya	27-Jan-2005	m	houses	line 1		KP339114
1586	Bantou	29-Jan-2005	f	houses	line 1		KP339115
1591	Bantou	29-Jan-2005	f	houses	line 1		KP339116
1605	Bantou	30-Jan-2005	m	houses	line 1		KP339117
1606	Bantou	30-Jan-2005	m	houses	line 1		KP339118
1645	Bantou	31-Jan-2005	m	houses	line 1		KP339119
1747	Bantou	18-Oct-2011	m	houses	line 1		KP339120
1755	Bantou	19-Oct-2011	f	maize	line 2	100 m	KP339121
1770	Bantou	19-Oct-2011	m	maize and ochra	line 5	30 m	KP339122
1786	Bantou	20-Oct-2011	m	houses	line 1		KP339123
1824	Bantou	21-Oct-2011	m	houses	line 1		KP339124
1826	Bantou	21-Oct-2011	m	houses	line 1		KP339125
1840	Bantou	21-Oct-2011	m	maize and ochra	line 5	30 m	KP339126
1858	Tanganya	23-Oct-2011	f	houses	line 1		KP339127
1873	Tanganya	23-Oct-2011	m	houses	line 1		KP339128
1878	Tanganya	23-Oct-2011	m	rice field dam	line 3	100 m	KP339129
1892	Tanganya	24-Oct-2011	f	houses	line 1		KP339130
1918	Tanganya	25-Oct-2011	f	houses	line 1		KP339131

1925	Tanganya	25-Oct-2011	f	houses	line 1		KP339132
1946	Tanganya	25-Oct-2011	f	maize and ochra	line 6	30 m	KP339133
Sok07	Sokourala	8-Nov-2013	m	houses	line 1		KT833175
Sok10	Sokourala	8-Nov-2013	m	houses	line 1		KT833176
Sok18	Sokourala	9-Nov-2013	m	houses	line 1		KT833177
Sok21	Sokourala	9-Nov-2013	f	houses	line 1		KT833178
Sok22	Sokourala	9-Nov-2013	m	houses	line 1		KT833179
Sok32	Sokourala	10-Nov-2013	m	houses	line 1		KT833180
Sok34	Sokourala	10-Nov-2013	m	houses	line 1		KT833181
Dam03	Damania	15-Nov-2013	m	houses	line 1		KT833182
Dam05	Damania	15-Nov-2013	m	houses	line 1		KT833183
Dam15	Damania	15-Nov-2013	f	houses	line 1		KT833184
Dam20	Damania	16-Nov-2013	f	houses	line 1		KT833185
Dam38	Damania	16-Nov-2013	f	houses	line 1		KT833186
Dam49	Damania	17-Nov-2013	f	houses	line 1		KT833187
Yar14	Yarawalia	20-Nov-2013	f	houses	line 1		KT833188
Yar20	Yarawalia	20-Nov-2013	m	houses	line 1		KT833189
Yar24	Yarawalia	21-Nov-2013	f	houses	line 1		KT833190
Yar36	Yarawalia	21-Nov-2013	f	houses	line 1		KT833191
Yar46	Yarawalia	21-Nov-2013	m	houses	line 1		KT833192
Kho10	Khoria	24-Nov-2013	f	houses	line 1		KT833193
Kho29	Khoria	26-Nov-2013	f	houses	line 1		KT833194
Kho30	Khoria	26-Nov-2013	m	houses	line 1		KT833195
Dal02	Dalafilani	28-Nov-2013	f	houses	line 1		KT833196
Dal04	Dalafilani	28-Nov-2013	m	houses	line 1		KT833197
Dal11	Dalafilani	28-Nov-2013	m	houses	line 1		KT833198
Dal17	Dalafilani	29-Nov-2013	f	houses	line 1		KT833199
Dal25	Dalafilani	29-Nov-2013	m	houses	line 1		KT833200
Dal29	Dalafilani	30-Nov-2013	f	houses	line 1		KT833201
Dal31	Dalafilani	30-Nov-2013	m	houses	line 1		KT833202
Saf01	Safrani	3-Dec-2013	f	houses	line 1		KT833203
Sil03	Silimi	6-Dec-2013	m	houses	line 1		KT833204
Bri08	Brissa	14-Dec-2013	m	houses	line 1		KT833205
Bri39	Brissa	16-Dec-2013	m	houses	line 1		KT833206
Dam71	Damania	26-Mar-2014	f	houses	line 1		KT833207
Dam72	Damania	26-Mar-2014	m	houses	line 1		KT833208
Dam90	Damania	27-Mar-2014	f	houses	line 1		KT833209
Dam95	Damania	27-Mar-2014	f	houses	line 1		KT833210
Sok40	Sokourala	3-Apr-2014	f	houses	line 1		KT833211
Sok44	Sokourala	3-Apr-2014	f	houses	line 1		KT833212
Sok51	Sokourala	3-Apr-2014	m	houses	line 1		KT833213
Sok61	Sokourala	4-Apr-2014	m	houses	line 1		KT833214
Sok62	Sokourala	4-Apr-2014	f	houses	line 1		KT833215
Sok75	Sokourala	5-Apr-2014	f	houses	line 1		KT833216
Sok76	Sokourala	5-Apr-2014	f	houses	line 1		KT833217
Sok77	Sokourala	5-Apr-2014	m	houses	line 1		KT833218

Son85	Sonkonia	8-Apr-2014	f	houses	line 1	KT833219
Son109	Sonkonia	9-Apr-2014	m	houses	line 1	KT833220
Yar51	Yarawalia	13-Apr-2014	f	houses	line 1	KT833221
Dal53	Dalafilani	19-Apr-2014	m	houses	line 1	KT833222

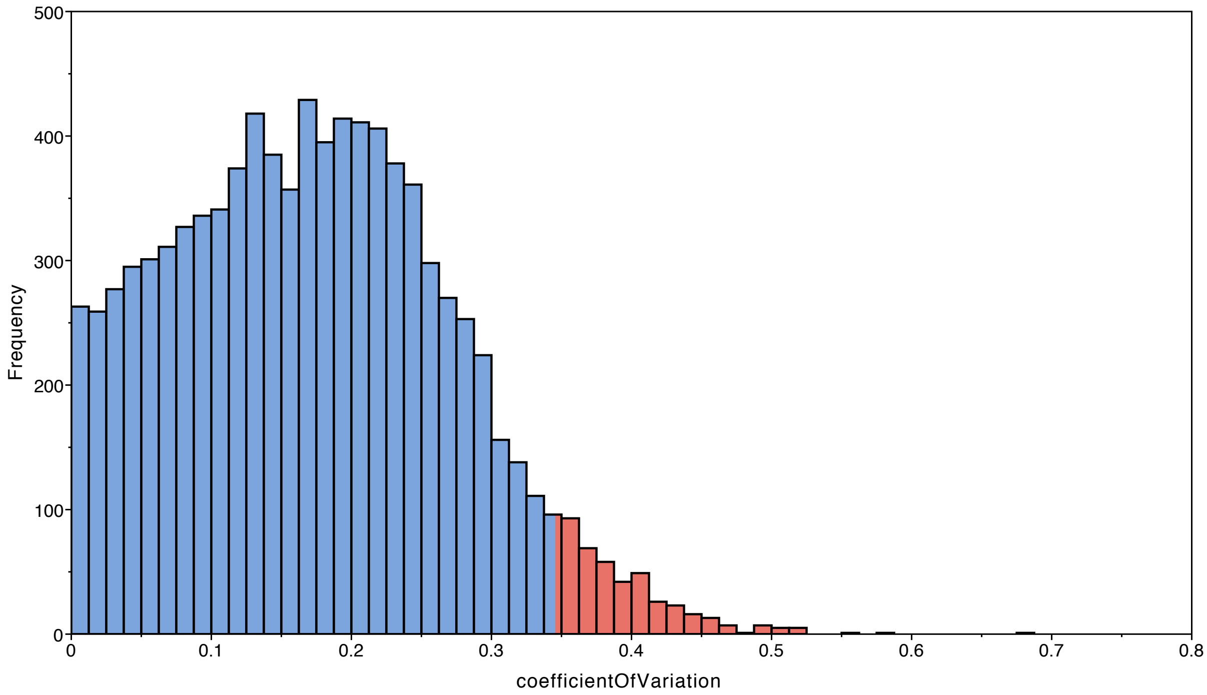

Figure S1: Frequency distribution of the coefficient of variation for model #3. The median is 0.13, indicating only minor differences in the molecular clock rate between branches.

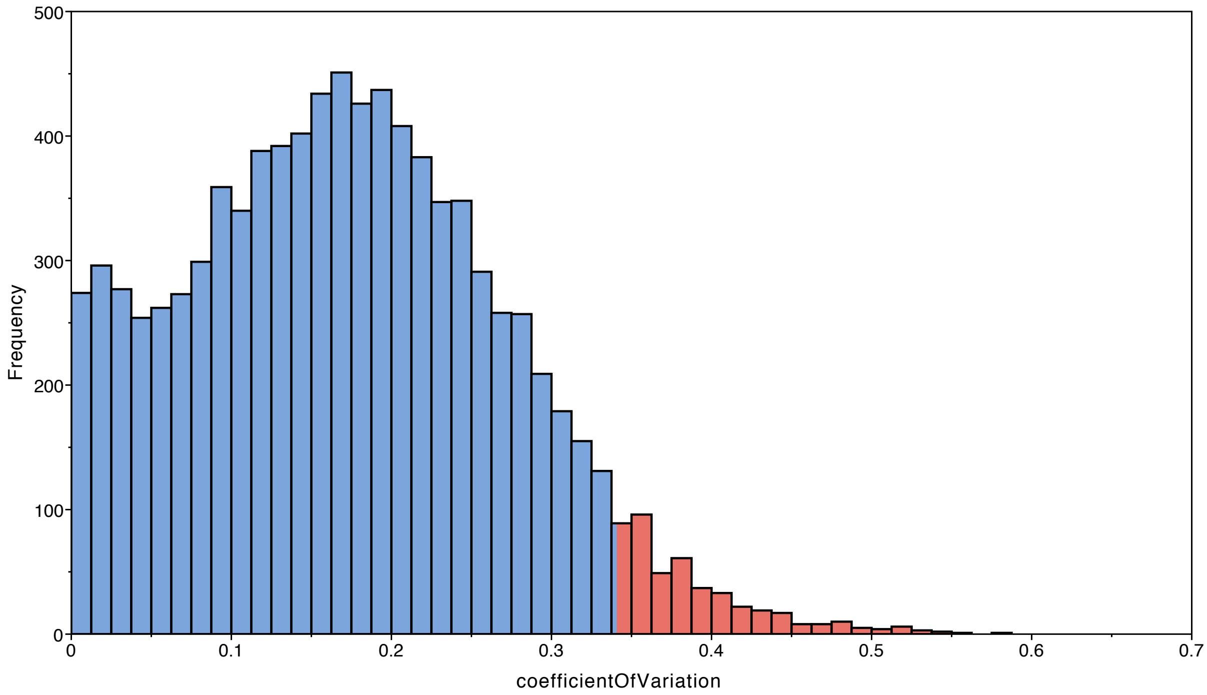

Figure S2: Frequency distribution of the coefficient of variation for model #4. The median is 0.13, indicating only minor differences in the molecular clock rate between branches.

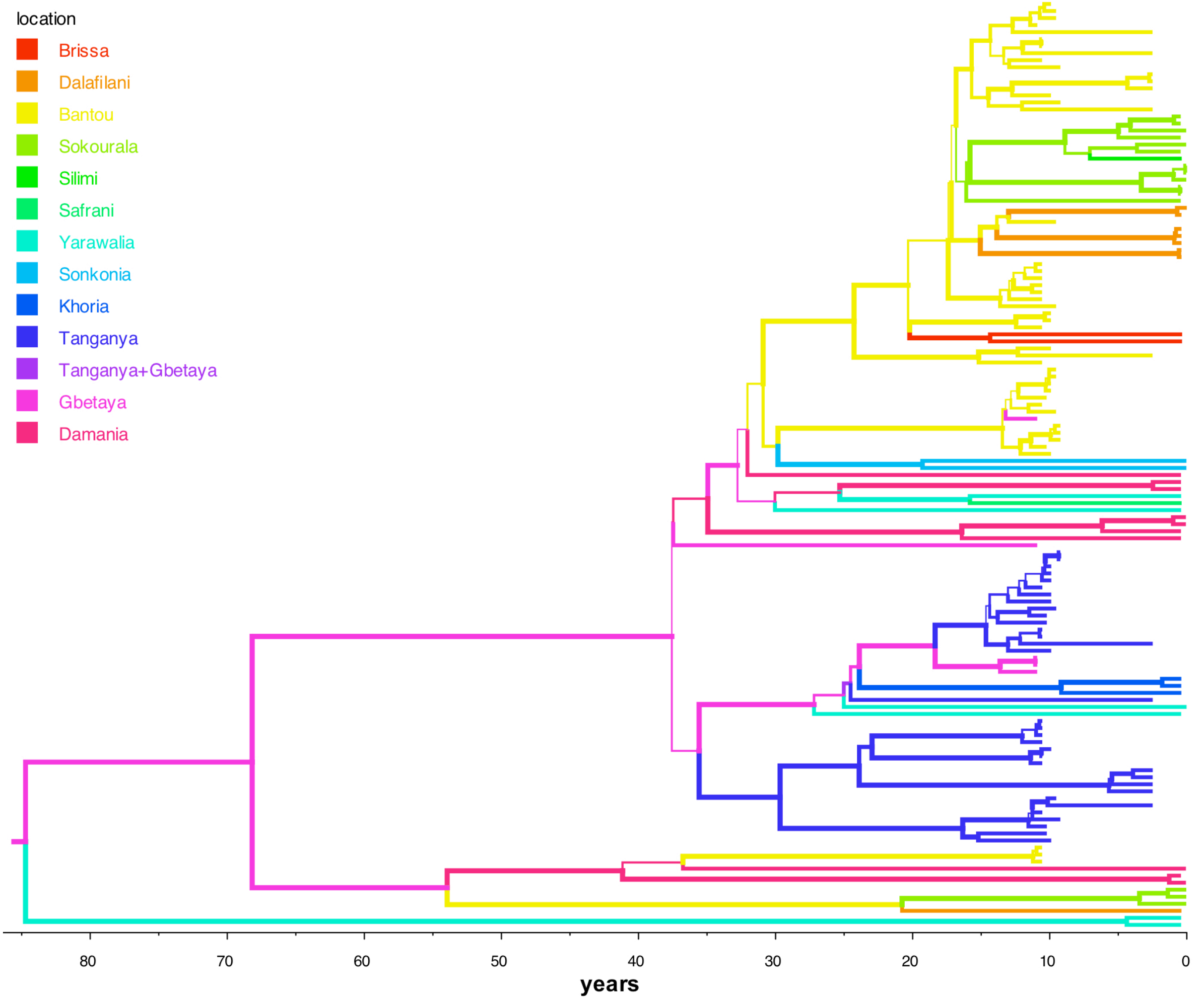

Figure S3: Phylogeographic analysis of LASV NP sequences from 132 *Mastomys natalensis* to visualise virus spead. The tree was inferred by using the Bayesian Markov Chain Monte Carlo method with GTR+gamma, strict clock, constant population size, and villages as traits. The villages are sorted by color gradient from North (Brissa) to South (Damania). Posterior support is indicated by the thickness of the branches (high values are represented by thick lines). Each change of color along the branches indicates virus spread from one village to another.
